# Cold Atmospheric Pressure Plasma Solutions for Sustainable Food Packaging

**DOI:** 10.3390/ijms25126638

**Published:** 2024-06-17

**Authors:** Azadeh Barjasteh, Neha Kaushik, Eun Ha Choi, Nagendra Kumar Kaushik

**Affiliations:** 1 Department of Physics, Lorestan University, Khorramabad 68151-44316, Iran; barjasteh.a@lu.ac.ir; 2 Department of Biotechnology, College of Engineering, The University of Suwon, Hwaseong 18323, Republic of Korea; neha.bioplasma@gmail.com; 3 Department of Electrical and Biological Physics, Plasma Bioscience Research Center, Kwangwoon University, Seoul 01897, Republic of Korea; ehchoi@kw.ac.kr

**Keywords:** cold atmospheric plasma, sterilization, bacterial inactivation, food packaging

## Abstract

Increasing the number of resistant bacteria resistant to treatment is one of the leading causes of death worldwide. These bacteria are created in wounds and injuries and can be transferred through hospital equipment. Various attempts have been made to treat these bacteria in recent years, such as using different drugs and new sterilization methods. However, some bacteria resist drugs, and other traditional methods cannot destroy them. In the meantime, various studies have shown that cold atmospheric plasma can kill these bacteria through different mechanisms, making cold plasma a promising tool to deactivate bacteria. This new technology can be effectively used in the food industry because it has the potential to inactivate microorganisms such as spores and microbial toxins and increase the wettability and printability of polymers to pack fresh and dried food. It can also increase the shelf life of food without leaving any residue or chemical effluent. This paper investigates cold plasma’s potential, advantages, and disadvantages in the food industry and sterilization.

## 1. Introduction

**Figure 1:**
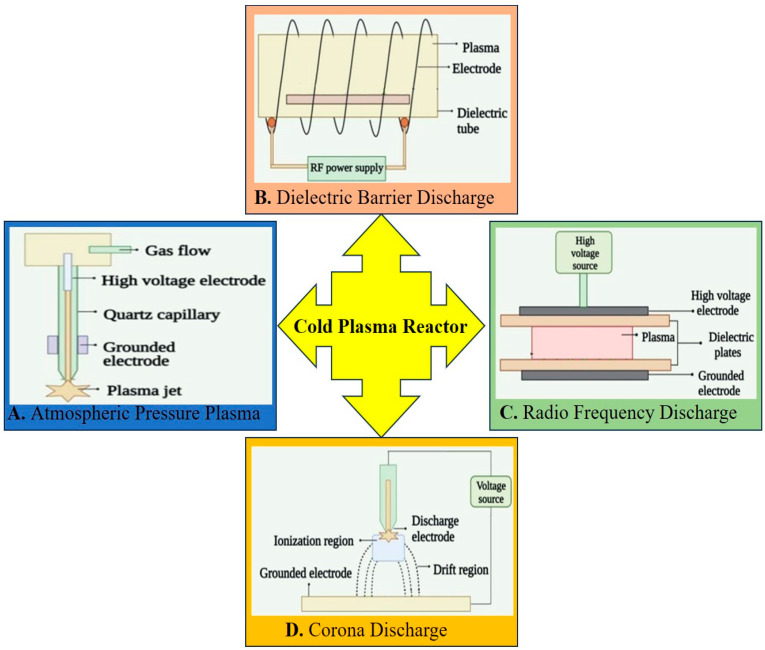
A schematic representation of different plasma reactors. (**A**) Atmospheric pressure plasma (APP), (**B**) dielectric barrier discharge (DBD), (**C**) radio frequency discharge (RFD), and (**D**) corona discharge [1].

The fourth state of the material, composed of electrons, ions, free radicals, excited species, ultraviolet (UV) radiation, and electromagnetic fields, is called plasma. It is produced when enough energy, like heat or electric power, is applied to natural gas. In the first division, plasma is divided into two groups: hot plasma and cold plasma. In high-pressure gas discharge, electrons and gas molecules collide frequently, resulting in thermal equilibrium between electrons and ions ($T_{e}=T_{ion}\geq 10^{4}\;K$). This type of plasma is called hot plasma. Still, in low-pressure gas discharge, electrons and ions do not collide frequently, and the electron temperature is so high that ions and, as a result, the gas temperature ($T_{e}\approx 10^{4}\;K\gg T_{ion}=T_{gas}=300\;K$) will remain at room temperature, which is called cold plasma. This process causes reactions that do not occur quickly in typical gases in cold plasma. Cold plasma can be produced in different forms, such as dielectric barrier discharge (DBD), glow or radiofrequency discharge, atmospheric pressure plasma (APP), and corona discharge. In their geometry, DBDs have at least one dielectric layer inserted between two electrodes where one is grounded and the other is connected to a high voltage. Dielectric materials cause charge accumulation and thus prevent arcing or sparking and cause the plasma to be cold. It is produced when low frequency (0.02 to 0.4 MHz), radio frequency (<500 MHz), or microwave (0.5 to a few GHz) is applied [1]. Radiofrequency discharge (RFD) is generally produced when a high pulse voltage is applied to a pair of electrodes, one of which is a pin-type corona discharge. Different types of cold atmospheric plasma (CAP) have been shown in Figure 1.

**Figure 2:**
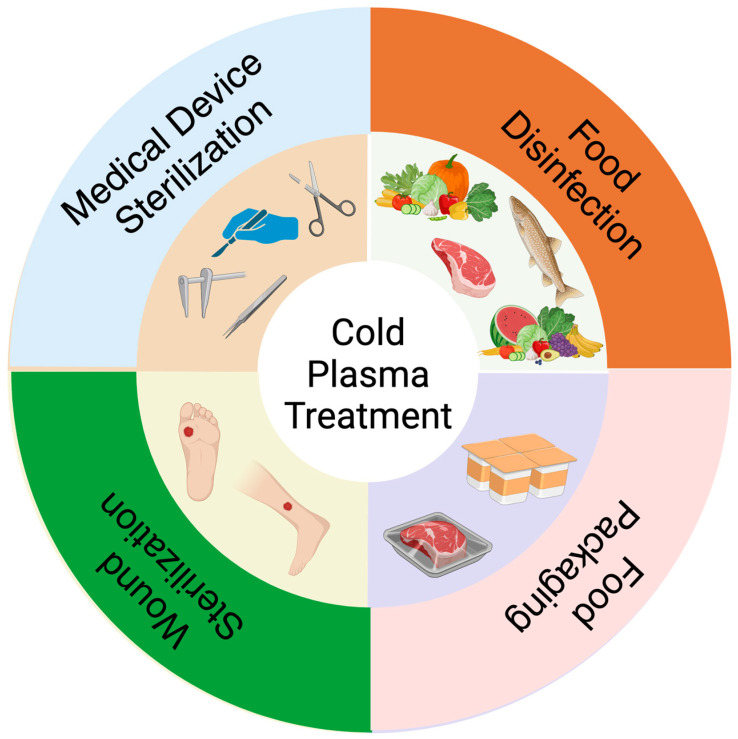
The current advances in cold plasma include wound sterilization, medical device sterilization, food disinfection, and packaging.

Different mechanisms in cold plasma are responsible for killing microorganisms, such as chemical reactions, electron bombardment, and UV radiation [2]. The chemical reaction efficacy depends on plasma composition and energy input, determining the density flow and plasma activation energy. Electron bombardment and UV radiation efficacy depend on the reactor’s electric field and radiation intensity, respectively. Nowadays, cold plasma is used in different applications such as wound sterilization [3,4], medical device sterilization [5], food, fruit, and vegetable disinfection [6,7], and food packaging [8,9], which has been shown schematically in Figure 2.

## 2. Current Advancements in Cold Plasma for Sterilization

Every year, thousands of deaths occur due to increasing bacteria that are resistant to treatment. Prion transmission from one patient to another through surgical devices is one of the most concerning medical fields. Different studies have shown that the prion proteins have a high affinity for the device structures, and conventional sterilization methods cannot remove them effectively [10,11]. Some of these devices, such as endoscopes, are expensive and reusable. Conventional sterilization methods include autoclaves, chemical gases, γ-radiation, and dry or wet heat. Evidence shows these conventional methods cannot remove all contamination or are expensive, such as γ- the radiation method, and their effects on material characteristics are unknown [12]. What reduces the drawback of these methods is that some polymeric devices are sensitive to heat and cannot be treated with autoclaves or wet or dry heat at a minimal temperature of 121 °C. On the other hand, some of these methods, such as chemical gases, use toxic gases like formaldehyde and ethylene oxide, which are harmful to the user and the environment [13,14]. Not only is there a resistance bacterial load in medical devices, but they are also present in different injuries such as diabetic foot ulcers and burning wounds that cause the death of millions of people all over the world each year. Because in these wounds, the skin loses its barrier against microbes, the pathogens that cause infections are introduced directly into the wound and cause infections that are resistant to antimicrobial drugs [15]. As a result, researchers are trying to find a way to effectively inactivate microbial loads and bacteria and prevent their growth, regardless of their kind. In this case, CAP can be an efficient therapy for treating burn wounds and diabetic foot ulcers [16]. In recent years, studies have shown that CAP is one of the most effective methods that can fight against pathogens, biofilms, and microorganisms [5,17,18,19,20]. Plasma reactive agents such as electrons, ions, free radicals, UV radiation, and electromagnetic fields caused by peroxidation of membrane lipids, oxidative damage to DNA, and acidification act against bacteria load [21] because the surface of microorganisms is composed of organic materials. The interaction of plasma with microorganisms destroys this organic layer [2], and then reactive particles in plasma reach the DNA of microorganisms to destroy them. Therefore, from one side, reactive species in plasma damage the cell wall and, on the other hand, damage the intracellular components such as nucleic acid, proteins, and DNA, and in this way kill the bacteria [22]. The efficacy of CAP treatment in deactivating bacteria and microorganisms depends on treatment time, distance to the sample, voltage and frequency, gas composition, and gas flow rate.

**Figure 3:**
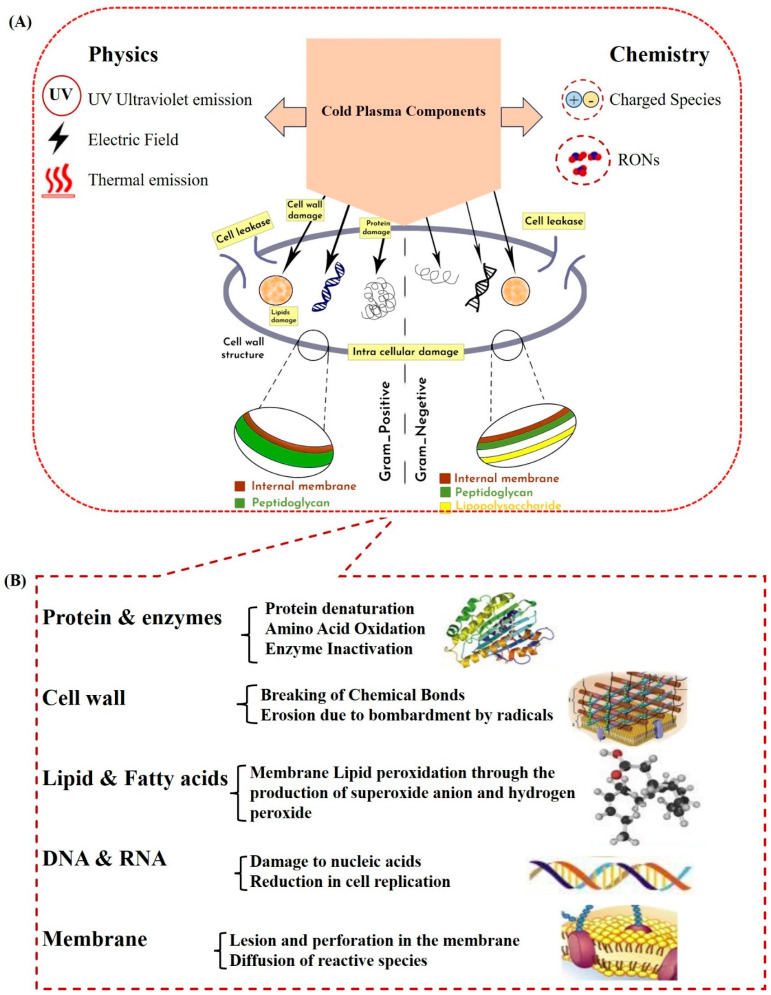
The physical and chemical effects of cold plasma on the Gram-positive and Gram-negative bacteria schematically (**A**) [29], and the mechanism of cold plasma’s effect on microorganism deactivation (**B**) [29].

The other factor that determines the efficacy of CAPs against bacteria is their peptidoglycan layer. Gram-positive and Gram-negative bacteria are two essential classes of bacteria based on their peptidoglycan layer. Gram-positive bacteria have thick peptidoglycan walls that cause them to resist plasma, and Gram-negative bacteria have thin peptidoglycan walls. Schematically, these two types of bacteria are shown in Figure 3A. As the thin membrane layer in Gram-negative bacteria is sensitive to ROS and UV radiation, CAP disrupts the outer membrane quickly and destroys the cell membrane [23]. As a result, intracellular compounds such as proteins, lipids, and DNA are released [24]. The Gram-positive bacteria do not have an outer membrane, but they have a peptidoglycan wall that ROS and UV radiation do not effectively damage. However, these reactive species can enter the cell through interstitial spaces and, in this way, destroy intracellular molecules [24,25]. As a result, CAPs damage the bacteria’s cell membrane, intracellular protein, and DNA, leading to the bacteria’s death [26]. In this case, the electrostatic field is due to charged particles in CAP that permeate the bacteria’s cell wall, leading to the breakage of chemical bonds, erosion, and openings in the membranes. Also, these charged particles cause the toxic compounds of plasma to enter the bacteria cell [27]. Therefore, the oxidative stress due to reactive oxygen radicals directly affects proteins and bacteria DNA [28]. Figure 3B schematically shows the mechanism of cold plasma’s effect on microorganism deactivation.

**Figure 4:**
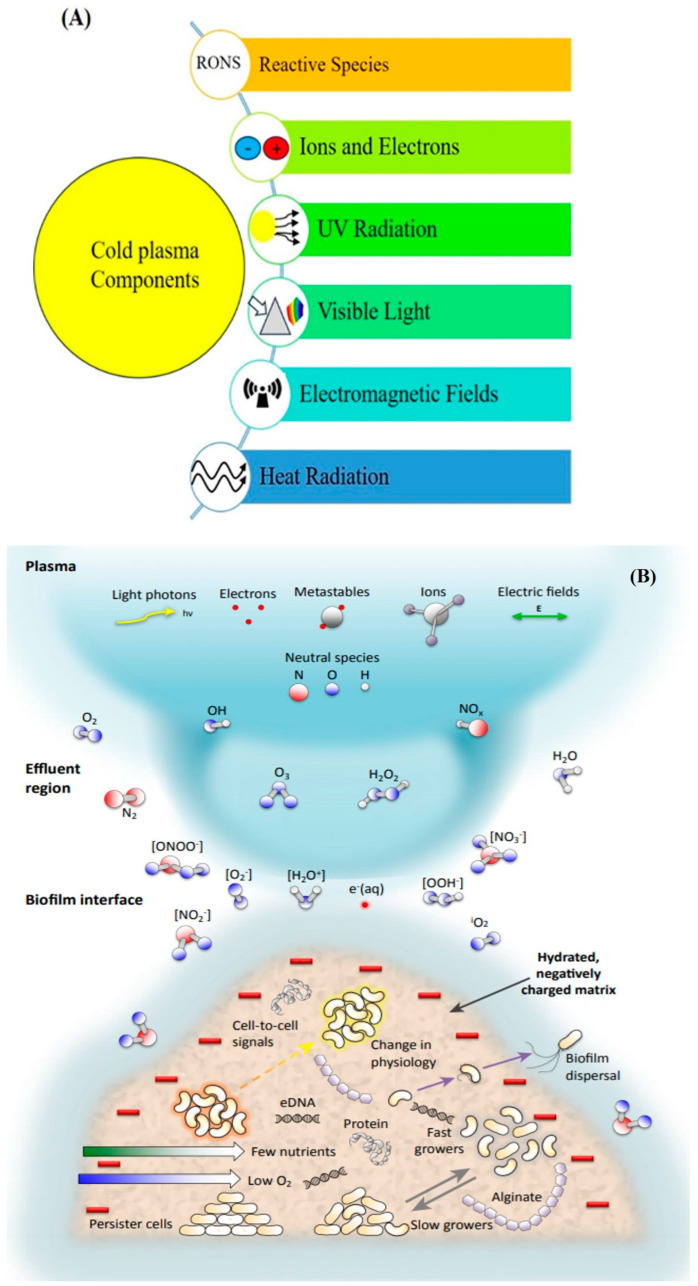
Cold plasma components (**A**) and the cold plasma’s effects on biofilms schematically (**B**) [30].

Another group of bacteria that is attached to a tissue matrix to absorb or produce extracellular polymeric substances (EPS) such as polysaccharides, phospholipids, and proteins [30] is biofilm. Because biofilms are resistant to harsh environments, most bacteria grow in community groups like biofilms [31]. Researchers have shown that the interaction of biofilms with cold plasma not only destroys biofilms through cell membrane destruction and damage to the intracellular substances, but it can also damage EPS, which is the base material for biofilm formation, interfere with quorum sensing (QS, which is a signaling molecule that can regulate intracellular communication), and in this way destruct biofilms [31,32,33]. The effects of cold plasma on biofilms are schematically shown in Figure 4.

### 2.1. Cold Atmospheric Plasma and Plasma-Activated Water: Two Different Ways of Using Plasma for Sterilization or Deactivation of Microorganisms

Plasma comprises reactive species such as electrons, ions, free radicals, UV radiation, and electromagnetic fields. When these species react with the water, they produce a cascade of chemical reactions. Through these chemical reactions, the water makes a mixture of the biochemically reactive medium called plasma-activated water (PAW) [34]. Some studies have shown that PAW can deactivate pathogens and bacteria effectively because of its chemically reactive mixture [12,22,34,35,36,37,38,39]. These studies have shown that the PAW’s inactivation efficacy is due to reactive oxygen species (ROS) such as OH, O_2_^−^, O_3_, and hydrogen peroxide (H_2_O_2_), and reactive nitrogen species (RNS) such as NO_2_^−^, NO_3_^−^, and ONOO^−^, which are dissolved in the water in the form of peroxynitrite (ONOOH) [40]. NO_2_^−^ and NO_3_^−^ between these reactive particles are classified as long-lived species, while OH and ONOO are classified as short-lived species. ONOO^−^ is a short-lived species but can penetrate the cell’s wall effectively and, as a result, shows a bactericidal effect [37].

Despite its short lifetime, OH is also very effective against bacteria [41]. The other factors of PAW are UV radiation, free electrons, and electromagnetic fields, which are present only during plasma discharge. Studies have shown that treating water with cold plasma increases the oxidation–reduction potential of treated water. At the same time, the pH of the treated water is decreased due to the formation of nitrous and nitrate acids as a result of the reaction of NOx species with the water molecules [42,43,44]. Nowadays, it is known that acidic solutions at low pH can effectively inactivate bacteria because of the penetration of reactive species into the cell wall [45]. Also, the bacteria are eradicated at a pH lower than 4.7 [45]. It has also been shown that a high level of oxidation–reduction potential, which is an indicator of ROS in PAW by changing the redox balance, increases the susceptibility of bacteria to an acidic environment [46]. As a result, a higher level of oxidation–reduction potential indicates that the ability of a solution to take electrons from the bacteria’s cell membrane is higher. Therefore, the cell will be unstable, causing the death of bacteria. As a ROS, peroxynitrite at low pH induces the permeation of the lipid bilayer in the cell membrane. In this way, more reactive oxygen nitrogen species (RONS) can penetrate the cell to initiate apoptosis and necrosis and, as a result, kill the cell [35]. It should be mentioned that PAW is transient and converts to plain water after some time. As a result, PAW is considered to be a green method to deactivate pathogens.

### 2.2. Cold Plasma and Inactivation of Microorganisms (Mechanisms and Factors Affecting) In Vitro and In Vivo

As mentioned previously, different studies have shown that CAP can inactivate microorganisms such as pathogens and biofilms [23,43,47]. The inactivation mechanism of CAPs is based on different reactive species such as ROS, RNS, charged particles, UV radiations, and electromagnetic fields in cold plasma [48,49,50]. In 2020, Gabrielle et al. investigated the efficacy of surface DBDs against bacteria such as *P. aeruginosa* in vitro and ex vivo in human skin cell models [16]. Their results showed that surface DBD is an efficient tool against bacteria both in vitro and ex vivo, so that it can effectively eliminate them in vitro in 2 min and ex vivo in 3 min. Different studies have shown that the shape of microorganisms will change when treated with CAP; for example, their membrane and cell wall will rupture, and the cell components will leak [51,52,53]. Other studies have shown that the cell membranes of microorganisms will lose their integrity with CAP treatment [54,55,56]. Different plasma agents have distinct roles in the deactivation of microorganisms in the plasma treatment of microorganisms. For example, the role of reactive species is to interact with microorganisms. The outer layer of microorganisms is composed of lipid materials, the main component of which is fatty acids. This layer has two prominent roles in microorganism maintenance: the first is that it acts against different agents as a defensive barrier [57,58], and the second is that it impedes protein molecules made of amino acids because they are susceptible to oxidation [57,58]. When treated, hydroxyl radicals (OH) and other RONS in cold plasma react with the lipid layer, leading to lipid peroxidation and aldehyde formation. Some studies have investigated the effect of CAP on DNA and other proteins in microorganisms [55,59,60]. Their results showed that CAP treatment leads to DNA destruction and protein degradation. As a result, RONS in cold plasma treatment is essential in destroying cell membranes and causing leakage of proteins outside the cell, which results in cell death. In recent years, some studies have investigated the effect of charged particles on the inactivation of bacteria [58,61,62]. Their results showed that charged particles, via electrostatic stress, facilitate microbial inactivation. The other factor in cold plasmas affecting microorganisms’ inactivation is UV radiation. Through photon absorption, this radiation starts photochemical reactions and, in this way, changes the molecular components of cell functions. In interaction with microorganisms, UV radiation penetrates the microorganism cell and reacts with intracellular components such as nucleic acid, which leads to cell death [63,64]. UV radiation generates interactions with intracellular components and reactive oxygen species that affect bacteria [58]. In this case, as indicated previously, they peroxidize the cell membrane and, in this way, damage the cell membrane. The role of the electromagnetic field in the plasma inactivation of bacteria has not been studied extensively. But what is evident is that in direct treatment, the effect of the electromagnetic field is more significant than in indirect therapy because, in direct treatment, the electromagnetic fields are produced in contact with the bacterial load, while in indirect treatment, plasma is first produced and blown out to the bacteria by the gas flow. As a result, in indirect treatment, there is a weak electromagnetic field in contact with the sample [65]. The mechanism of cold plasma’s effect on microorganism deactivation is schematically shown in Figure 3B.

**Figure 5:**
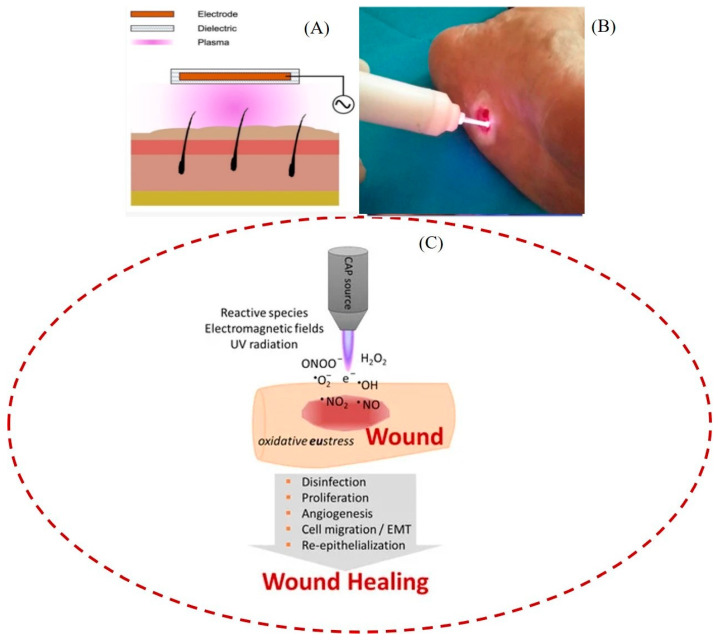
Direct treatment (**A**) [71] and indirect treatment of wounds (**B**) [72]; the effect of cold plasma on wound healing (**C**) [4].

The presence of bacteria such as *P. aeruginosa* and *S. aureus* in wounds, such as burn wounds, causes delayed healing and more extended hospitalization [66]. The current treatments, such as antibiotics, are not enough to eliminate these bacteria, and as a result, resistance to antibiotics has increased. Therefore, other treatments should be applied. Also, biological disinfectant is so tricky. In recent years, CAPs have shown that they can be effective against clinical pathogens and drug-resistant bacteria [67,68,69,70]. Because they have different geometries, they can easily be applied to biological surfaces. Plasma can be used on biological surfaces in two different ways: direct and indirect methods, which are schematically shown in Figure 5A,B. In the direct method, plasma is produced in contact with the skin; in this case, the skin acts as the second electrode, and in the indirect method, plasma is made between two electrodes and then moves to the wound side by the gas flow [69,70].

Not only device design but also treatment time, gas composition, distance from the sample, and voltage and frequency of applied voltage are essential factors in the bactericidal effect of CAP on wound sterilization. The environment of the sample, such as wound type, is another critical factor in the bactericidal effect of CAP because wound exudate and biofilm formation, which are environmental factors of the wound, affect the efficacy of CAP. In this case, bacteria can resist CAP treatment not only in biofilms but also in wound exudate, which can increase pH, which causes a reduction in CAP efficacy against bacteria [73,74]. Studies have shown that in sterilizing wounds, the direct application of CAPs acts more effectively than the indirect ones [69] because, in the indirect method, some reactive species that have a short lifetime will disappear before reaching the wound site. In the plasma treatment of wounds, different plasma agents, such as reactive species, UV radiation, and electromagnetic fields, affect the wound differently. Different studies have investigated the effect of reactive species on wound sterilization. They have shown that in plasma treatment, there are different RONS such as O, O_2_, O_3_, OH, H_2_O_2_, NO, NO_2_, and NO_3_. These reactive species, in contact with the microbes, damage their membranes to deactivate them [75]. In between all of these reactive species, O_3_ is the most effective species for deactivating bacteria by oxidizing their cell walls [76]. UV radiations via photochemical reactions penetrate the microorganism’s genetic material and cause cell death [64]. Not only can plasma destroy microorganisms in the wound, but it can also stimulate wound healing using blood vessel formation and proliferation via its reactive species and electromagnetic fields [77]. Figure 5C schematically shows the effect of cold plasma on wound healing. To investigate the impact of CAP on the reduction in bacterial loads in diabetic foot ulcers (DFU), Peters et al. (2017) applied a volume DBD to 20 patients [78]. Their results showed that plasma can effectively reduce the bacterial load of *S. aureus*. In another study in 2020, Buke et al. investigated the efficacy of CAP treatment in deactivating bacteria both in vitro and in healthy volunteers [3]. Their results showed that plasma treatment in a short time can reduce bacterial load effectively, and it is a safe treatment because it does not cause DNA damage or reduce cellular activity.

### 2.3. Medical Device Sterilization

Some medical devices, like endoscopes and colonoscopes, are relatively expensive, and we must reuse them for diagnoses and surgery. Some of these devices cannot be autoclaved because they are sensitive to temperature. Conventional disinfecting methods include rinsing, brushing, and disinfection using an automated preprocessor. Different studies have shown that these methods are not enough to disinfect them, and some infections will remain in them [79,80,81]. Therefore, researchers have investigated other methods, such as using ethylene oxide to disinfect them. These methods are time-consuming, harmful to users and the environment, and have financial costs [82,83]. In recent years, studies have shown that CAPs can disinfect these plastic devices without producing excessive heat, the need for rinsing, harmful effects on personnel, or environmental residue [58,84]. Different studies have indicated that CAPs, through various mechanisms, can help disinfect these medical devices [18,84]. The first mechanism is gas plasma flow, which can partly remove the physically absorbed soil and proteins like amyloids, either physically or through chemical reactions [18]. The other mechanisms are performed by using RONS, as described above.

**Figure 6:**
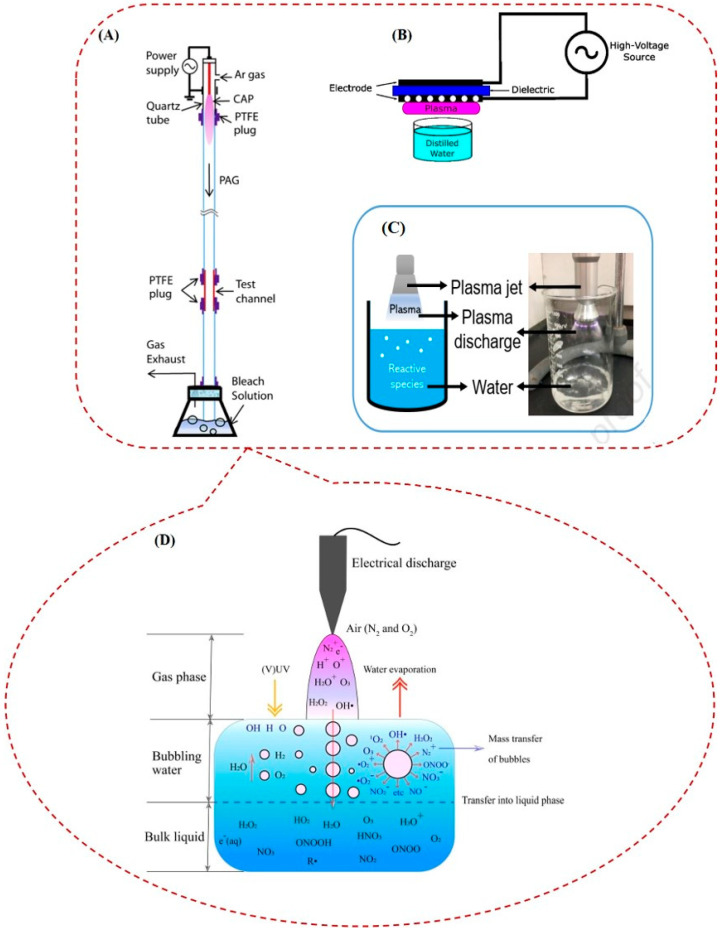
Plasma-activated gas to disinfect the contaminated channels, such as endoscope tubes. (**A**) [84], direct (**B**) [12], and indirect plasma-activated water (PAW) to disinfect medical devices (**C**) [89], and different agents made in PAW (**D**) [90].

Studies have shown that these species effectively deactivate bacterial biofilms through acidification and increase oxidation–reduction potential [85,86]. Increasing oxidation–reduction potential can damage the cell membrane of bacteria and oxidize the cellular glutathione [35,87]. Plasma for sterilization of medical devices is used in direct and indirect forms; the indirect form includes plasma-activated gas (PAG) and plasma-activated water (PAW) to disinfect medical devices [84]. The plasma plume will be confined inside the medical devices in PAG, which are usually used to disinfect contaminated channels like endoscope tubes. Figure 6A shows a type of PAG. Like in PAW, plasma will not be produced directly in contact with the sample in PAGs; therefore, some reactive species with a short lifetime will be lost before reaching the sample. However, studies have shown that using PAG is a safe technology that can eradicate drug-resistant bacteria, biofilms, and bacteria spores [84,88].

PAW is produced when plasma is in contact with water. In this case, plasma first changes the water to an acidic liquid by reducing pH, and this acidic liquid is effective against bacteria [91,92]. The central effectiveness of PAW is due to its acidic properties and RONS production. Figure 6B,C show, respectively, the surface DBD and plasma jet that are used to produce PAW. Because of some events, such as species transfer and chemical reactions between reactive species of plasma and water, some reactive species, such as O_3_, O_2_^−^, and H_2_O_2_, are produced [93]. Not only reactive oxygen species but also reactive nitrogen species such as nitric oxide (NO), peroxynitrite, and NO_2_^−^ are made in contact with the plasma in the water to produce a complex environment in PAW that has an antibacterial and cytotoxic effect [94] and is schematically shown in Figure 6D.

## 3. Plasma and Food Industry

In the food industry, maintenance and increasing food materials’ shelf life are crucial in food packaging because pathogenic microorganisms and microbial toxins can spread through food materials and cause infections. These days, different methods, such as sterilization, canning, drying, steaming, frying, cooking, and backing, which are thermal processes, are used for preserving food materials [95]. While they are very customary, they significantly reduce the quality of food materials regarding vitamins, proteins, and nutrients [96]. The other methods for preserving and storing food material are freezing and pasteurization, thawing, and UV radiation, which can effectively inactivate microorganisms. Despite their effectiveness in maintaining food material, some pathogenic foodborne bacteria, such as Clostridium and Bacillus, can survive in inactive spore forms [97]. These microbial spores are resistant to post-processing treatments remaining in the processed products and are the main reason for foodborne disease and intoxication. Due to these disadvantages, different nonthermal methods such as ultrasonication, irradiation, high-pressure processing, and pulsed electric fields have been extended that can inactivate microorganisms and microbial spores. These new nonthermal methods improve nutrient retention and inhibit microorganism growth [98].

The most important limitation of the industrialization of these new technologies is their high cost. Hence, finding a proper way to preserve food materials such as fruits, vegetables, and agricultural products is a critical factor in the food industry. Nowadays, different materials such as plastics, polyethylene materials like polyethylene terephthalate (PET), HDPE, and LDPE, polyvinyl chloride, metals like tin, aluminum, and steel, glass, cardboard, ceramic, and cellulose are extensively used in food packaging to reduce microbial activities and increase the shelf life of fresh food materials such as fruits and vegetables [99]. Besides their advantages, there are disadvantages, such as high production costs and limited size, and some break easily, like glass. In addition, the production of these methods can release carbon dioxide, which is harmful to the environment. Therefore, researchers investigate other packaging materials like bio/polymeric nanocomposites made from lipids, proteins, and polysaccharides to overcome these problems [100,101,102]. One of these bio/polymeric materials is chitosan, which is used effectively for perishable fruit packaging. Different studies have shown that this material is not reinforced enough for the postharvest process [8,103], while it is a natural biodegradable and antibacterial material. Therefore, different methods, like contaminating them with cellulose materials and adding chemical materials, are used to improve them [104,105]. One of the essential methods that has shown promising results in the food industry is CAP, which can deactivate microorganisms and microbial spores and reinforce nanocomposites at a low cost [7,106]. The reactive species in cold plasma can also change the properties of unsuitable biopolymers, such as wettability, printability, and mechanical properties, and create desired polymers for food packaging [107,108], while having no thermal effect on them because they work at room temperature.

### 3.1. Cold Plasma Treatment of Food Packaging Material Surface

**Figure 7:**
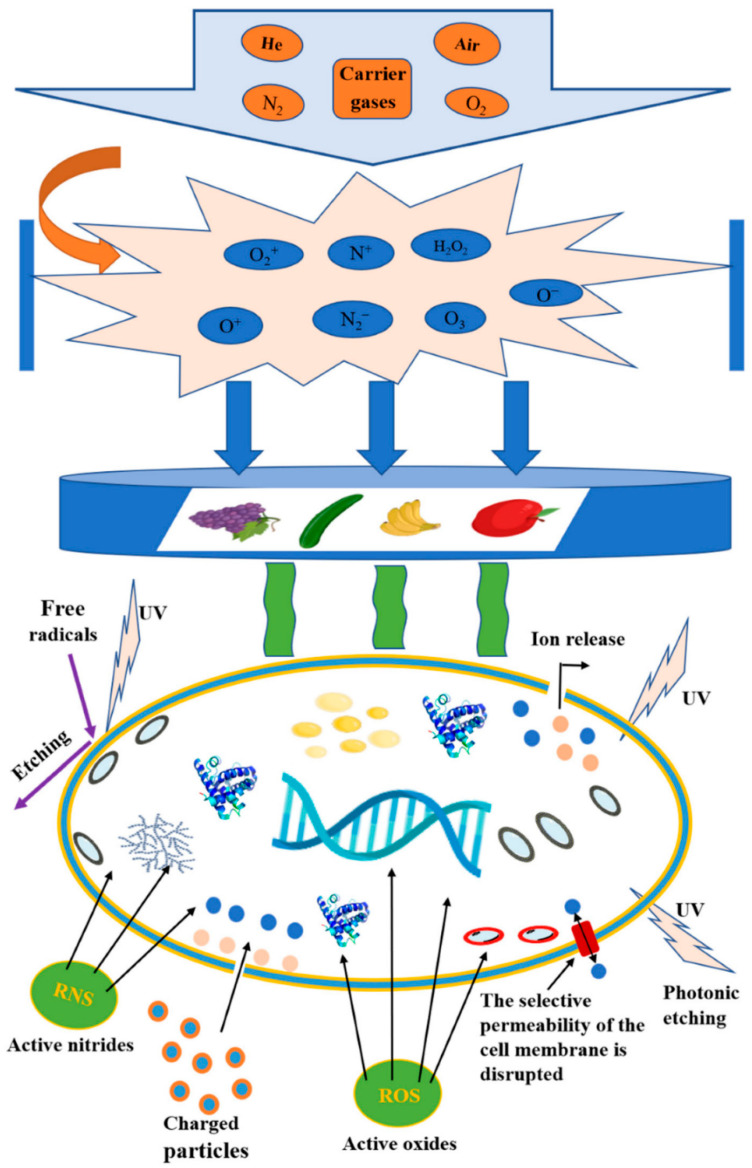
The action mechanism of cold plasma on the surface of food materials [111].

Two crucial processes determine whether the food industry is thriving: (i) sterilization of food packaging material and (ii) preservation of packed food [109]. As the manufacturing process’s final stage is packaging, removing any contamination introduced into the product or packaging materials requires disinfection or sterilization. Also, the key factors that determine a product’s shelf life are the decontamination of that product and the sterilization of its packaging material [9]. Packaging prevents environmental decontamination from entering the product during distribution and is also used to preserve food quality. If the packaging material has not been appropriately sterilized, contamination from the packaging surface enters the food, leading to health risks. Therefore, different treatments, such as thermal treatment, UV radiation, and chemicals like ethylene oxide and hydrogen peroxide, are used to sterilize these packaging materials. However, these methods have different drawbacks, such as liquid effluents and increasing costs [9]. Because thermal treatment of fresh food products for decontamination affects nutritional and textural quality, novel treatment methods such as cold plasma are needed. Also, since heat treatment destroys some food packaging materials, such as their film-like materials, cold plasma can also be a new treatment for food packaging materials. The by-products of plasma treatment of food products or food packaging materials are CO_2_, water, and carbon monoxide in small amounts dependent on plasma active species that, in comparison with 10 min of automobile exhaust, are equivalent to 1 year of sterilization by cold plasma [9,110]. As a result, cold plasma is a clean, safe, and green technology in the food industry without chemical residue. Figure 7 schematically shows the action mechanism of cold plasma on the surface of food materials.

**Figure 8:**
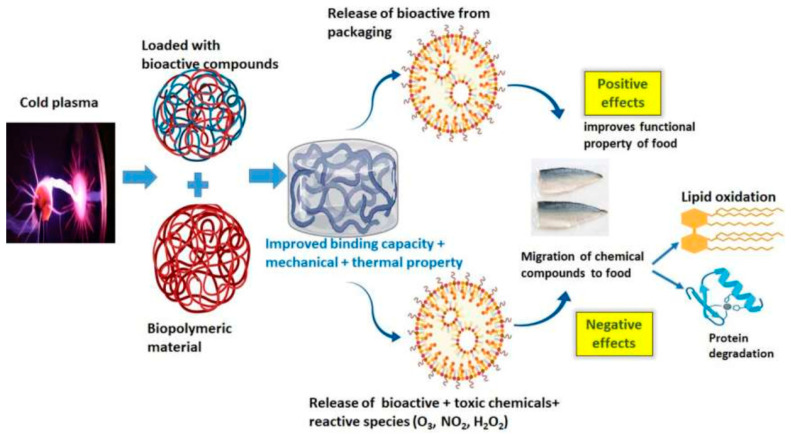
The schematic representation of the effect of CAP on food packaging materials [117].

As previously mentioned, in two ways—directly and indirectly—cold plasma can be used to decontaminate food materials. Different studies have shown that the best type of plasma in the food industry is DBD because its operating and maintenance costs are low and the reactive species in it are higher than other types of cold plasma [112,113]. In these cases, the polymeric package acts as a dielectric layer, and the microbial population of a food product decreases due to the direct effect of reactive species. In recent years, different studies have investigated the impact of DBDs on different food packaging materials, such as PET, polyethylene (PE), polystyrene (PS), nylon, and paper foils [114,115,116]. Figure 8 shows a schematic representation of the effect of CAP on food packaging materials.

Polymers are a significant contributor to all packaging materials because of their benefits, such as low cost, low weight, transparency, and ease of use. Still, they are hydrophobic and have low surface energy, which is unfavorable to the food industry regarding weak adhesion, printing, and dyeing [118]. Different studies have shown that cold plasma treatment does not change polymeric food packaging materials’ physical and chemical properties. Still, it can remove some bioactive functional compounds such as lysozyme, vanillin, and specific peptides on the surface of packaging material, which are microbially resistant [9,119,120]. It has also been shown that using cold plasma can deposit some antimicrobial compounds, such as Auranta FV and Nisin, on the surface of polyethylene packaging materials to increase their antimicrobial resistance against bacteria, yeast, and molds and increase the shelf life of foods [121,122]. Other studies have shown that cold plasma treatment can physically increase the surface roughness of polymeric packaging materials due to the bombardment of plasma reactive species and chemically change the surface composition of polymer material via attachment of reactive species to the polymer chains and, as a result, modify them in terms of improved wettability, printability, and salability without changing their pulk properties [123,124]. Cold plasma can also sterilize food packaging materials by interacting reactive species with microorganism cells adhered to polymer surfaces without any residue [115]. In this case, reactive species in cold plasma and UV radiation damage the DNA of bacterial cells to eradicate them.

### 3.2. Disadvantages of Cold Plasma Technology on Food Products

Although cold plasma has numerous advantages in food processing, it can cause mild alternations, such as unpleasant physical and chemical changes like changes in color and lipid oxidation, respectively, that can finally lead to economic loss and health concerns and negatively affect shelf life [97,125]. For example, in 2012, Fröhling et al. showed that plasma treatment causes the color of pork meat to change to green due to reactions of myoglobin to H_2_O_2_ present in cold plasma [126]. Lipid oxidation is also caused by free radicals such as H_2_O_2_ and O_2_ ions that may lead to deoxy myoglobin and oxymyoglobin oxidation by removing hydrogen atoms from lipid atoms [127,128]. This effect is especially evident in meat products such as pork, beef, chicken, seafood, and sushi [129]. It has also been shown that the oxidation effect occurs in meat products and cereal products such as white rice, brown rice, and wheat flour [6]. To reduce the oxidation effect due to CAP treatment, some studies have suggested selecting a proper carrier gas composition and using antioxidant compounds such as essential oil to reduce lipid oxidation during plasma treatment [130,131,132]. Another significant drawback of using cold plasma treatment is that cold plasma cannot wholly deactivate the enzymes, producing enzymatic browning and surface degradation such as polyphenol oxidases and peroxidases [133]. In juices, it has been shown that cold plasma treatment via ozonolysis causes the degradation of polymerized oligosaccharides [134]. In the case of biopolymer-based films, it has been shown that CAP treatment can cause thermal property changes [135,136,137]. In this case, Pankaj et al. (2014) showed that plasma treatment of sodium caseinate films caused a decrease in the glass transition temperature of the films due to chemical etching [137]. Therefore, in some cases, plasma reduces the thermal stability of biopolymer-based films, which is one of the main problems with using them. About fruits, it has been shown that cold plasma leads to acidification and discoloration of fruits and also decreases firmness [138]. As a result, treatment with cold plasma causes acidification, discoloration, and unfavorable changes in tissue characteristics. In this case, it has been shown that the negative changes due to cold plasma treatment can be minimized via optimization of plasma parameters such as power input, feeding gas, gas flow rate, treatment time, and voltage and frequency of applied voltage. For example, it has been shown that plasma treatment of chicken fillets for short treatment times, such as 3 and 9 min, does not change the color, while 10 min of plasma treatment changes the color of chicken breast [139,140,141]. Another disadvantage of cold plasma treatment is the aging effect, whereby the impact of cold plasma on packaging surfaces is not permanent because dynamic processes occur due to the reduction in free surface enthalpy [142]. For example, the hydrophilicity created by cold plasma treatment in polymer layers is reduced due to inward diffusion, aggregation, and reorientation of polymer chains.

### 3.3. Safety of Cold Plasma for Food Products

One of the main problems regarding food products is foodborne disease and intoxication, which are caused by microbial spores. Therefore, novel technologies such as cold plasma treatment [7], ultrasonication [143], and pulsed electric fields [144] have been proposed to reduce these foodborne microorganisms. Cold plasma has different advantages, from microorganism deactivation to increasing the shelf life of fresh foods without using any chemical material. These advantages caused cold plasma to be considered a green technology in the food industry. In recent years, different studies have been conducted to investigate the toxicity of cold plasma in the treatment of foods because, in cold plasma treatment, reactive species are in direct contact with food surfaces. Some of these studies showed that cold plasma treatment has no toxicity effect on food products [145,146]. For example, in a study in 2016, Kim et al. investigated the toxicity of plasma water treated as a nitrite source for sausage meat [145]. Their analysis showed no immunological toxicity or mutagenicity in mice fed with them. In another study, Han et al. investigated the safety of edible films treated with cold plasma [147]. Their results also showed no evidence of toxicity in animal models.

Regarding wastewater, it has been shown that CAP treatment can reduce the amount of contamination [148]. Another essential advantage of cold plasma treatment is its ability to inactivate spores and microbial toxins [106,149,150]. Also, one of the main critical safety concerns of food products is the formation of biofilms on food contact surfaces, which is a primary microbial survival mechanism and is often resistant to food preservative treatment and sanitizers. Different studies have shown that cold plasma treatment has a promising effect against these biofilms because reactive plasma species both penetrate the biofilm cells and damage their structural integrity [151,152]. Regarding food packaging, some packaging materials, such as PET, cannot withstand heat. Therefore, cold plasma can be applied because of its effectiveness in eliminating cross-contaminants during the packaging process of food materials at low temperatures. It has also been shown that plasma can alter the structure of cellulose compounds in edible films, which in turn causes their better breakdown and, as a result, improves them for use in food packaging [153].

Regulatory bodies play a crucial role in ensuring the safety and quality of products in both the healthcare and food industries. Regulations and standardization of nonthermal plasma devices, processes, and products are urgently required for all industrial applications. Regulations must cover healthcare, food packaging, and other industrial processes separately. General standard regulations relevant to plasma industries are currently being developed by organizations such as IEC, ISO, ASTM, DIN, CE, and others. In healthcare settings, medical regulatory bodies from specific countries collaborate to establish unified international standards with other relevant authorities. On the other hand, food safety authorities, together with other regional authorities, are preparing regulatory guidelines for food packaging based on plasma processes to confirm safe and efficient processes in the industry.

## 4. Conclusions

This review summarizes the effect of CAP as an efficient sterilizer for the decontamination of microorganisms in both food products and food packaging materials. Plasma does its decontamination effects via RONS and UV radiation while at ambient temperature and has no thermal effect on food or food packaging material. Also, different studies have shown that plasma with short treatment times, such as less than 4 min, is safe for humans because it eliminates toxic effects due to reducing pH or heat, does not cause inflammation or DNA damage, and can destroy bacteria effectively. Plasma is created using functional groups on polymers such as PET or PP to increase surface energy, wettability, and printability, thus improving their packaging for food products. It has also been shown that plasma can annihilate toxins caused by foodborne microorganisms. Despite these advantages, they have disadvantages such as unpleasant physical and chemical changes such as color change and oxidation of lipids, reduction in thermal stability of polymers, acidification and color change in fruits, and instability of the effects created on fruits, which has caused this technology not to be approved by the US FDA for use in the food industry. Some of the details on the CAP applications in various healthcare and food industries have been mentioned in Table 1.

## Figures and Tables

**Table 1:** A table that outlines the applications of nonthermal plasma across various industries, including healthcare and food packaging.

Industry	Application	Advantages	Disadvantages
Healthcare	Surface sterilization of clinical equipment	Effective against various pathogens, non-toxic process	Moderate material degradation, high cost
	Wound healing application	Accelerates tissue regeneration, reduces infection	Limited clinical studies
	Cancer treatment	Selective on cancers, non-toxic process	Limited clinical studies
	Dental application	Effective in disinfection, non-invasive	Equipment cost requires skilled staff
Food Packaging	Increasing shelf life	Cold method, maintain nutritional quality	Less penetration in dense materials
	Surface sterilization	Reduces waste, increases safety	Equipment cost and maintenance
	Active packaging	Improves quality of food, enhances preservation, enhances shelf life	Material compatibility and cost
Textiles	Surface modification	Environmentally friendly process, maintain material quality	Cost and maintenance of equipment
	Microbial inactivation/resistant	Long-term effects, green process	Slight material degradation
Electronics	Surface cleaning	Removes contaminants, improves adhesion	Cost and equipment complex process
	Etching and patterning	High precision, ecofriendly	Specificity and process rate
Agriculture	Abiotic/biotic Stress removal	Ecofriendly process enhances immunity	Specific equipment or processes required
	Seed treatment	Improves germination, disease resistance	Variability in outcome
	Agri-products sterilization	Decreases contamination, enhances shelf life	Regulatory issues
Environmental	Air purification	Eradicate pollutants, decreases odors/bad smell	Power usage and maintenance
	Water treatment	Efficient decontamination, Ecofriendly green process	Energy consumption and equipment cost
	Soil remediation	Sterilize contaminants, improve quality, eco-friendly	Variable effectiveness
Cosmetics	Skin treatment	Promotes wound healing, reduces oxidative stress, reduces acne, activates skin	Limited clinical studies
	Anti-aging treatments	Enhance collagen synthesis, reduces senescence	Specified skin product required for market
Pharmaceutical	Drug delivery system	Maintain drug quality, targeted drug delivery	Regulatory challenges
	Packaging sterilization	Ensures decontamination, cold and green method	Setup cost and maintenance
